# Worldwide mapping of initiatives that integrate population cohorts

**DOI:** 10.3389/fpubh.2022.964086

**Published:** 2022-10-03

**Authors:** Laura Alejandra Rico-Uribe, Daniel Morillo-Cuadrado, Ángel Rodríguez-Laso, Ellen Vorstenbosch, Andreas J. Weser, Laura Fincias, Yannick Marcon, Leocadio Rodriguez-Mañas, Josep María Haro, José Luis Ayuso-Mateos

**Affiliations:** ^1^CIBERSAM (Network-Based Biomedical Research Consortium, Area of Mental Health), Instituto de Salud Carlos III, Spanish Ministry of Science and Innovation, Madrid, Spain; ^2^School of Health Sciences, Universidad Internacional de La Rioja, Logroño, Spain; ^3^Instituto de Investigación Sanitaria La Princesa (IIS-LP), Hospital Universitario de La Princesa, Madrid, Spain; ^4^CIBERFES (Network-Based Biomedical Research Consortium, Area of Frailty and Healthy Ageing), Instituto de Salud Carlos III, Spanish Ministry of Science and Innovation, Madrid, Spain; ^5^Parc Sanitari Sant Joan de Déu, Barcelona, Spain; ^6^Department of Medicine, Universitat de Barcelona, Barcelona, Spain; ^7^HUNT (The Trøndelag Health Study) Research Centre, Norwegian University of Science and Technology, Trondheim, Norway; ^8^Epigeny, St. Ouen, France; ^9^Department of Psychiatry, Universidad Autónoma de Madrid, Madrid, Spain

**Keywords:** cohort studies, harmonization, integration, mapping, population cohorts

## Introduction

There has been a recent surge in scientific data collection in the healthcare domain (1). Several large-scale cohort studies have recently been or are currently being conducted around the globe: the Millennium Cohort Study (2), the Environmental Health Risks in European Birth Cohorts (3), the Collaborative Research on Aging in Europe (4), the English Longitudinal Study of Aging (5), and the Survey of Health, Aging and Retirement in Europe (6) are popular examples. The *integration* of these individual cohorts—i.e., combining similar data from different studies into a unified whole—is the next step to getting the most valuable information out of them. Cohort integration studies may increase sample sizes drastically, allowing the study of important but infrequent phenomena (e.g., rare diseases) (7) while avoiding publication bias. The pressure is rapidly building up to keep up to date with integrating and capitalizing on this information, and several initiatives have joint forces for integrating cohorts from different studies [e.g., ATHLOS (8); BioSHaRE (9); CHICOS (10); HELIX (11)].

Despite this, there are concerns about legal (i.e., data protection) and ethical aspects, as well as methodological and infrastructure challenges (12), with lack of interoperability being the most prominent one. All of these may hinder the potential benefits of cohort integration studies. Overcoming these difficulties implies the harmonization of the different cohort data, and performing aggregated analyses on the integrated cohorts. *Harmonization* refers to practices aimed at improving the comparability of variables by reducing heterogeneity across studies (13), and is a necessary step for cohort integration. Harmonization can be *prospective* (studies share the same study design and measurement instruments from their inception), *ex-ante retrospective* (studies were not originally designed to be comparable but use standard collection tools and operating procedures) or *ex-post retrospective* (when no common standard formats or protocols are used across studies) (14). Regarding the analyses, three options are available: *pooled analysis* combines all the data from individual participants into a single analysis by aggregating them into a common data infrastructure; *meta-analysis* is a two-stage analysis that combines the results of inferences on each separate cohort dataset (15); *federated analysis* performs the analysis of individual level data in a central infrastructure while participant-level data remain on their local infrastructure.

An overview of how researchers are applying those methods to overcome the challenges is still missing in the scientific landscape. Systematizing this knowledge may help further integrate existing cohorts, as well as streamline the design and integration of future cohorts. In an attempt to expand and make these efforts more systematic, the European Commission called for a sustainable, strategic agenda for a better, global coordination of cohorts. The *SYNergies for Cohorts in Health: integrating the ROle of all Stakeholders* (SYNCHROS) project was funded by the Horizon 2020 Research and Innovation Program with the goal of formulating this strategy, through intensive stakeholder collaboration (https://www.synchros.eu). Among others, one of its first actions was the mapping of the cohort integration initiatives landscape across the world and in Europe especially. The objective was to obtain first-hand information about the methodologies and solutions implemented for integrating patient, clinical-trial, and population cohorts.

The patient cohort landscape mapping has already been reported elsewhere (16). This paper focuses on the state of the art in the integration of population cohorts. We present the methodology for gathering a collection of initiatives that have integrated such cohorts in the last 20 years, hereby defining *initiative* as any project (regardless of the number of byproduct publications) that has analyzed and/or plans to analyze data from different cohorts in an integrated manner, in order to draw conclusions from these data altogether (in contrast to accumulating evidence by analyzing the data from each cohort individually). Additionally, we show the results and conclusions of this mapping exercise.

## Materials and methods

### Identification of initiatives

Three different methods were used to find initiatives that integrated population cohorts. The first one was a systematic search in the MEDLINE database. The second one consisted of asking the SYNCHROS consortium and scientific officers of the European Commission for suggestions on initiatives. The third was a descendent search using the information (references, descriptions, and links) from the two previous sources.

### Database search in MEDLINE

The search was intended to produce a representative, albeit non-exhaustive, list of cohort integration initiatives. An initial set of search terms was agreed upon by the consortium partners. In order to accomplish a manageable number of hits, these terms were tested and the ones that yielded more than 500 hits were discarded. (To reduce the number of hits, the term “cohort” was added to some searches before the discarding). New terms were extracted from abstracts of relevant retrieved papers and tested again. To obtain the most recent scientific evidence, the search was limited to studies published in 2000 or later. The search was conducted in July 2019. The final search query was:

(cohort OR
“prospective study” OR
“longitudinal study” OR
“individual meta-analysis”[All Fields] OR
“individual participant data meta-analysis”[All Fields] OR
“individual patient data meta-analysis”[All Fields] OR
“individual meta analysis”[All Fields] OR
“individual participant data meta analysis”[All Fields] OR
“individual patient data meta analysis”[All Fields] OR
“meta analysis using individual”[All Fields] OR
“meta-analysis using individual”[All Fields] OR
“meta analysis of individual”[All Fields] OR
“meta-analysis of individual”[All Fields] OR
“mega-analysis”[All Fields] OR
“mega analysis”[All Fields])
AND
(“harmonization study” OR
“integration study” OR
“integration initiative” OR
“integrated study” OR
“merged cohort” OR
“data pooling” OR
“pooled sample” OR
“combined data” OR
“combining data” OR
“harmonized data” OR
“harmonized data” OR
“harmonizing data” OR
“data harmonization” OR
“data harmonization” OR
“data sharing” OR
“common database” OR
“multiple cohorts” OR
“multiple longitudinal studies” OR
“international consortium” OR
“collaborative effort”).
AND
(“2000/01/01”[Date–Publication]: “2019/07/31”[Date–Publication])
AND
English[Language]
AND
Humans[MeSH]

### Selection of initiatives

#### Inclusion criteria

Initiatives published in English from 2000 to July 2019 were included if they integrated health population cohorts of any age (birth, adolescents, adults, elderly, oldest old), and included sociodemographic, lifestyle, biological, genetic, omics (genomics, proteomics, metabolomics), imaging, or environment factors data. The SYNCHROS project has a focus on the coordination of longitudinal cohort studies due to their validity for determining causal relationships. Therefore, at least one of the integrated cohorts was required to have information about the sample at two time points at least.

#### Exclusion criteria

Initiatives without available data about (or access to) their descriptive information (webpage, a main report describing the main aim(s) in detail) were excluded. Initiatives that integrated clinical trial cohorts and/or patient cohorts are the subject of another publication (16) and thus were also excluded.

**Figure 1:**
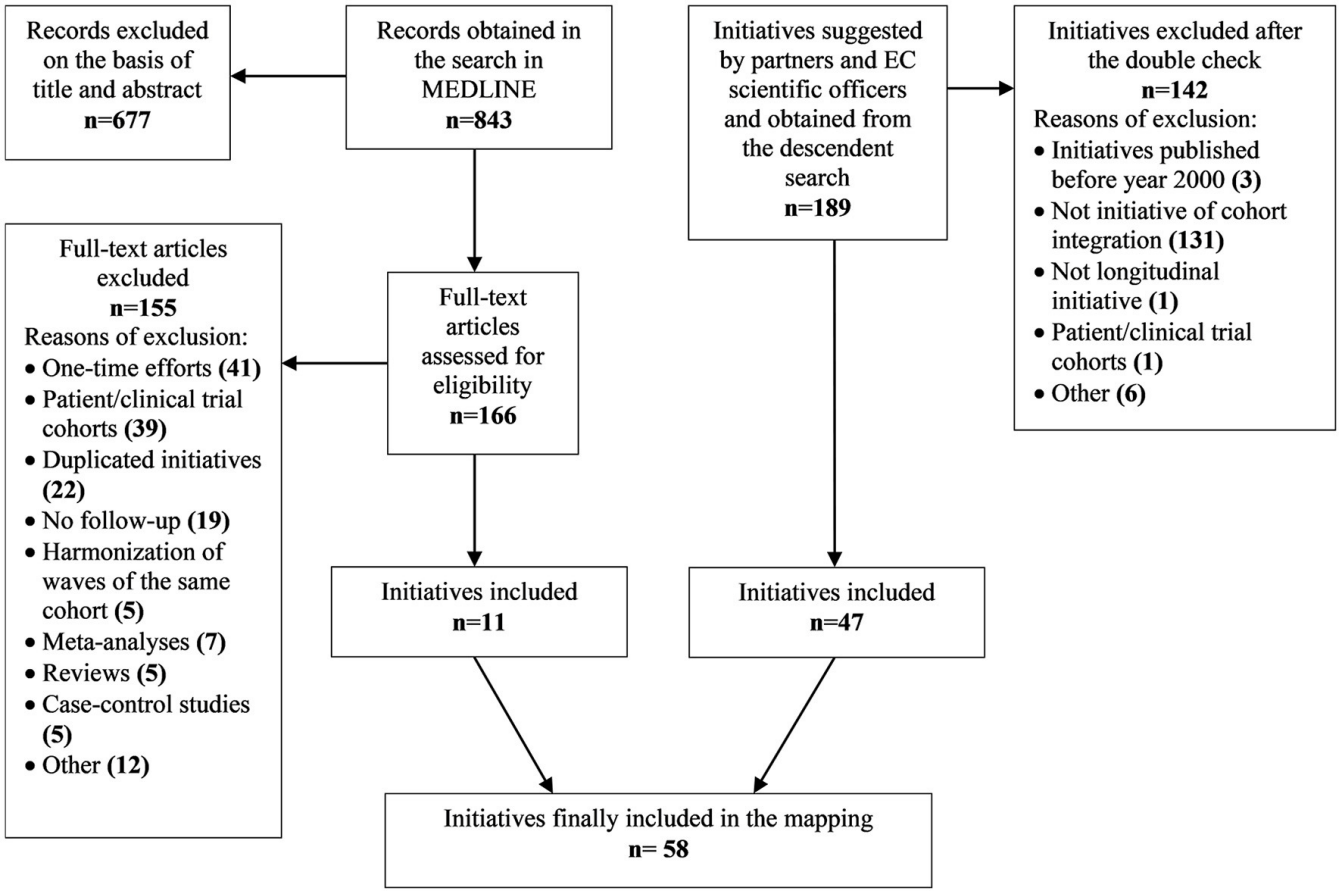
Flowchart diagram detailing the initiatives included and excluded.

For all the initiatives found, a double-check was performed by two different researchers in order to carry out an objective evaluation and reduce the risk of bias. In case of discrepancy between the two reviewers, a third person was consulted. The flowchart diagram in Figure 1 summarizes the initiatives included and excluded using the three methods.

The MEDLINE search identified 843 articles that described initiatives to be potentially included. After screening the titles and abstracts, 166 articles met the inclusion criteria. From the full-text review, 155 articles were excluded due to one or more of the following reasons: one-time efforts (i.e., data merged *ad-hoc* for the specific analysis supporting the paper), initiatives already submitted by partners, articles with cross-sectional harmonization only, multisite cohorts/harmonization of waves, meta-analyses, reviews, case-control studies, patients' cohorts, clinical trial cohorts, and others. From the systematic review, 11 articles were included. Additionally, from the partners' and officers' suggestions and the descendent searches, 189 potential initiatives were obtained. After screening for eligibility, 141 projects were excluded for one or more of the following reasons: initiatives published before the year 2000, projects that did not integrate cohorts, one initiative with only cross-sectional cohorts, and one initiative that integrated patient and clinical trial cohorts. This resulted in the inclusion of another 47 initiatives that integrate population cohorts. Thus, the total of initiatives included amounted to 58.

### Data extraction

The following technical information was extracted from the initiatives: name, principal investigator (PI), initiative partners, name of the leading institution, contact person, information source, whether the research team was active at the time of consultation, main objectives, criteria for the cohorts to be included, funding resources (public, private, or mixed public/private), and a brief description of the population addressed. When the information was not available through the website or published articles, the principal investigator and/or the contact person (i.e., any person that could respond to the form requesting information about the initiative) of the project was consulted, by email first and, if no reply was received, by telephone or postal mail.

The following information about the harmonization process was collected: harmonization strategy (prospective/retrospective ex-ante/retrospective ex-post), number of harmonized cohorts, whether more cohorts were foreseen to be harmonized, number of participants with harmonized data, maximum number of harmonized variables (including those where harmonization was not possible for all the cohorts), and setting of the harmonized cohorts (local-regional/national/international). The following information was deemed relevant for the integration effort and thus was also collected for each initiative: total number of cohorts, total number of participants, age range of the sample, country/ies included, whether metadata and individual data were accessible to other researchers, whether any of the cohorts included biological (omics) samples, research topic(s) addressed, and type(s) of aggregated analyses performed on the integrated cohorts (pooled, federated, or meta-analysis). The initiatives are presented in Supplementary Table 1, including a subset of their most representative information.

The country where the leading institution was based and the countries included in the integrated cohorts were recoded into continents (Africa, Asia, Europe, Oceania, Latin America & Caribbean, North America). Descriptive statistics were computed for all the outcomes when appropriate: absolute frequencies and proportions (over number of non-missing values) for the categorical ones; median, minimum, and maximum for the quantitative ones. In the case of the number of participants with harmonized data and the maximum number of harmonized variables, the statistics were computed using only the initiatives with harmonized cohorts. In the case of the age range of the sample, some initiatives did not have concrete numerical values and were not taken into account.

## Dataset descriptive statistics

In a large number of initiatives the contact person was unreachable throughout the whole mapping process, so the proportion of missing data was as large as 70.7% for some variables. At the time of consultation, 83.3% of the research teams that had carried out the projects remained active (although this percentage drops to 69.0% if we assume that those which did not respond were inactive). The majority of the leading institutions were based in The Netherlands (*n* = 9), followed by institutions from the USA (*n* = 8), the UK (*n* = 6), and Finland (*n* = 5). Of the initiatives, 82.6% were funded by public institutions, while 17.4% received funding from both private and public institutions. Interestingly, none of the three initiatives addressing the “Birth, infancy & childhood health” received funding from private institutions, while the two initiatives that reported studying cancer did receive funding from them. Although these numbers are too low to draw safe conclusions, it might seem that the research interests of the private sector are biased toward certain health topics while neglecting others.

The total number of cohorts ranged between three and 84 (median = 14). Some initiatives had up to 69 harmonized cohorts, while others had only collected the participating cohorts (but had not started harmonizing them yet). At least 22 initiatives planned to obtain and harmonize more cohorts in the future. The harmonized cohorts had a large variability in the maximum number of harmonized variables, ranging from 12 to 37,000 (median = 270). They would comprise up to 26,62,777 participants, although the median value was around 169,000. Ex-post (retrospective) harmonization was by far the most prevalent strategy, used by 75.5% of the initiatives. The majority of initiatives (86.0%) used pooled analysis to perform integrated analysis on the cohorts. Meta-analysis and federated analysis were less common, being used by only 30.2% and 25.6% of the initiatives respectively (note that some initiatives used more than one type of integrated analysis).

The targeted populations ranged in age from childbirth to death (the 150-year-old upper limit probably refers to a cohort with unbounded maximum age, rather than participants of that age). Aging was the most prevalent topic, addressed by 15.5% of the initiatives, and 42.1% (*n* = 8) of the initiatives included omics data (i.e., biological samples). The cohorts were collected in as many as 43 countries, although there were also initiatives with cohorts from only one country. Most of them (51.8%) were circumscribed to just one continent, with Europe being the most frequent (80.4%), while only 3.6% comprised cohorts from all six continents. Latin America and Africa were the most underrepresented continents, with only six (11.3%) and nine (16.1%) initiatives including cohorts from them. The complete descriptive information of the initiatives can be found in Table 1. Furthermore, the most up-to-date information on each initiative can be consulted in the repository of the SYNCHROS project (https://repository.synchros.eu).

**Table 1:** Descriptive statistics of the initiatives mapped.

**Variable**	**Level**	**Median/*n* (*N* = 58)**	**(Range/%)**
Team active (at consultation)		40	(83.3%)
	(Missing)	10	
Continent of leading institution	Africa	1	(2.0%)
	Asia	3	(5.9%)
	Europe	32	(62.7%)
	Oceania	4	(7.8%)
	Latin America & Caribbean	0	(0.0%)
	North America	11	(21.6%)
	(Missing)	7	
Funding	Public	38	(82.6%)
	Private	0	(0.0%)
	Mixed public/private	8	(17.4%)
	(Missing)	12	
Access to metadata	Yes	17	(51.5%)
	No	2	(6.1%)
	Under request	14	(42.4%)
	(Missing)	25	
Access to individual data	Yes	3	(17.6%)
	No	5	(29.4%)
	Under request	9	(52.9%)
	(Missing)	41	
Topic	Chronic diseases	4	(6.9%)
	Cancer	3	(5.2%)
	Cardiovascular diseases	4	(6.9%)
	Musculoskeletal diseases	2	(3.4%)
	Neurological diseases	3	(5.2%)
	Respiratory diseases	1	(1.7%)
	Communicable diseases	1	(1.7%)
	Biomedicine	1	(1.7%)
	General epidemiology	7	(12.1%)
	Public health	5	(8.6%)
	Aging	9	(15.5%)
	Birth, infancy & childhood health	4	(6.9%)
	Mental health	4	(6.9%)
	Environmental health	7	(12.1%)
	Social environment	5	(8.6%)
	Occupational health	2	(3.4%)
	Reproductive health	1	(1.7%)
	Medical imaging	2	(3.4%)
	Genetics	6	(10.3%)
	Genomics	1	(1.7%)
	Other omics	3	(5.2%)
With omics data		8	(42.1%)
	(Missing)	39	
No of cohorts	Total	14	(3–84)
	(Missing)	10	
	With harmonized data	8	(0–69)
	(Missing)	17	
More cohorts foreseen to be harmonized?		22	(50.0%)
	(Missing)	14	
No of harmonized variables (maximum)		270	(12–000)
	(Missing)	35	
No of participants	Total	2,45,000	(700–2,10,00,000)
	(Missing)	17	
	With harmonized data	1,69,000	(700–26,62,777)
	(Missing)	34	
Age	Minimum	18	(0–95)
	(Missing)	24	
	Maximum	90	(12–150)
	(Missing)	35	
No of countries		5	(1–43)
	(Missing)	26	
Setting	International	39	(73.6%)
	Local/regional	1	(1.9%)
	National	13	(24.5%)
	(Missing)	5	
Continent	Africa	9	(16.1%)
	(Missing)	2	
	Asia	16	(28.6%)
	(Missing)	2	
	Europe	45	(80.4%)
	(Missing)	2	
	Oceania	15	(26.8%)
	(Missing)	2	
	Latin America & Caribbean	6	(11.3%)
	(Missing)	5	
	North America	24	(45.3%)
	(Missing)	5	
Harmonization strategy	Ex-ante	8	(15.1%)
	Ex-post	40	(75.5%)
	Prospective	5	(9.4%)
	(Missing)	5	
Analysis	Federated	11	(25.6%)
	Meta	13	(30.2%)
	Pooled	37	(86.0%)
	(Missing)	15	

## Conclusion

The purpose of this mapping was to gather knowledge about the state of the art of the initiatives that integrate population cohorts. The landscape of these initiatives has been revealed to be quite disparate, with a high variability of populations and variables, as well as topics and regions addressed by the integrated cohorts. As foreseeable, the number of participants and variables integrated is generally larger than in initiatives integrating patient cohorts (16). Taking the high volumes of data in some of the initiatives, their potential for future researchers is undeniable. Nevertheless, the number of integration initiatives seems rather low, compared to the overall and increasing number of very large cohort datasets worldwide. It is also worth stressing that a large proportion of the initiatives found are inactive nowadays, limiting the possibilities of data access and sharing.

Most of the initiatives found were funded by public entities (and mainly by the European Commission). We found a scarcity of private funding, with only a few initiatives being partially funded by private institutions. One might think that, for example, pharmaceutical companies would be highly interested in integrating evidence across cohorts and thrive toward personalized medicine, especially in patient and clinical trial cohorts. However, the participation of private entities in funding patient cohorts was even lower (16). Nevertheless, it should be noted that these funding sources refer only to the integration initiatives; this information does not take into account other possible funding sources for the individual cohort studies integrated into an initiative, so private interest in funding cohort research might be overlooked.

Focus on environmental exposures was more prevalent than genetic and biological factors. Very few of the initiatives were actually found to harmonize and integrate biological sample data (although there was a high rate of missingness in this variable). Including them may help discover possible causal pathways among biological, behavioral, social, demographic, economic, and health outcomes (17). Unfortunately, incorporating bio-measures in cohort research is not always feasible.

Most of the initiatives were led by European and American institutions; as would be expected, most of the integrated cohorts were also collected in these two continents. Interestingly, very few of the initiatives included cohorts focused on African and Latin American & Caribbean countries. Previous studies emphasize the relative lack of health studies conducted in low- and middle-income countries [LMICs (18)]. More representativeness of LMICs would be necessary to grant the external validity of the cohort studies that inform global health policy recommendations.

### Strengths and weaknesses

To our knowledge, this is the first effort to map and describe in detail all the initiatives integrating population cohorts. Given the difficulties reported by authors when integrating cohorts (19), we expect positive outcomes of our endeavor in three main aspects. First, the SYNCHROS repository is a resource where interested researchers can find integrated population cohort data or contribute their population cohorts. Second, several PIs and project managers have provided first-hand information on the barriers and solutions they have found when integrating cohorts. Finally, we expect all this information to be extremely relevant to designing a European strategy for cohort integration, such as is the aim of the SYNCHROS project.

Although we must raise awareness of the non-exhaustive nature of this mapping, we should stress that this manuscript aims at representativeness rather than exhaustiveness. It should be noted though that, given the huge amount of work involved in integrating population cohorts, we deem it unlikely that we have missed any initiative that has published results. Furthermore, as the response rate of the principal investigators was rather low, relevant information on some initiatives is still missing at the time of submission of this manuscript. However, the SYNCHROS repository (https://repository.synchros.eu) is an evolving project, where the most recent information available is continuously updated.

### Final remarks

Knowledge about restricted populations and phenomena (e.g., personalized medicine, rare diseases, epigenetics) requires massive sample sizes to achieve the necessary statistical power. Moreover, global representativeness can only be achieved by addressing more diverse populations (20) from different ethnic, regional, and/or socioeconomic settings. Synergies across a wide variety of existing cohort integration projects would help pursue these goals, while being more cost-effective than undertaking new international mega-cohorts (21). Dedicating efforts to designing cohort studies with the challenges of cohort integration in mind would be highly recommendable. Otherwise, these integration initiatives may ultimately lead to a greater resource expenditure than new cohort studies themselves.

However, there is still a shortfall of initiatives that integrate worldwide population cohorts. Most of the currently available cohorts probably lack the necessary transparency and availability of information (variables, study designs, data access, etc.) to afford multi-study research. There are excellent examples of best-practice principles of integration of both patient and population cohorts, such as Dementias Platform UK (https://www.dementiasplatform.uk), and the Integrative Analysis of Longitudinal Studies of Aging (IALSA) network (https://www.ialsa.org). We expect the SYNCHROS repository to be an additional valuable resource for emerging collaborative research, with a spotlight in developing and enriching a “learning healthcare system” (22).

## Data availability statement

The original contributions presented in the study are publicly available. This data can be found here: https://github.com/CCOMS-UAM/mapping-initiatives/blob/main/dat/initiatives-dataset.xlsx.

## Author contributions

LR-U contributed to the acquisition, analysis, and interpretation of data and wrote the first draft of the manuscript. DM-C analyzed and interpreted the data and drafted the subsequent versions of the manuscript. ÁR-L contributed substantially to the conception and design of the study and the data collection survey, participated in the acquisition, analysis and interpretation of the data and in the writing of the manuscript, and revised it critically. EV contributed to the conception and design of the study, the acquisition, analysis and interpretation of data, and the writing of the manuscript and its critical revision. AW contributed to the acquisition, analysis, and interpretation of data, as well as to the writing of the manuscript and its critical revision. LF contributed to the data acquisition and revised the manuscript critically. YM contributed to the design of the data collection and deployment of the SYNCHROS repository, collaborated with the data acquisition, and revised the manuscript critically. LR-M, JH, and JA-M contributed to the conception and design of the study, and revised the manuscript critically. All authors approved the final version of the manuscript and agree to be accountable for all aspects of the work.

## Funding

The results reported herein correspond to specific aims of grant 825884 to the SYNCHROS Consortium from the European Union's Horizon 2020 research and innovation program. LR-U's and DM-C's work was supported by the Network-Based Biomedical Research Consortium, Area of Mental Health (CIBERSAM), Instituto de Salud Carlos III, Spanish Ministry of Science and Innovation.

## Conflict of interest

The authors declare that the research was conducted in the absence of any commercial or financial relationships that could be construed as a potential conflict of interest.

## Publisher's note

All claims expressed in this article are solely those of the authors and do not necessarily represent those of their affiliated organizations, or those of the publisher, the editors and the reviewers. Any product that may be evaluated in this article, or claim that may be made by its manufacturer, is not guaranteed or endorsed by the publisher.

## Supplementary material

Supplementary Table 1 Main information of the initiatives included in the mapping.
