# Supplementary material for: Worldwide mapping of initiatives that integrate population cohorts
Source: Front Public Health. 2022 Oct 3;10:964086. doi: 10.3389/fpubh.2022.964086 (PMC9574101; doi:10.3389/fpubh.2022.964086)
Supplement: Supplementary Table 1 — Main information of the initiatives included in the mapping. [file Data_Sheet_1.ZIP › Supplementary_Table_1.html]

Supplementary Table 1


# Supplementary Table 1

Main information of the initiatives included in the mapping.  
*Note:*
ARG = Argentina; AUS = Australia; AUT = Austria; BEL = Belgium; BGD = Bangladesh; BRA = Brazil; CAN = Canada; CHE = Switzerland; CHL = Chile; CHN = China; CRI = Costa Rica; CUB = Cuba; CYP = Cyprus; CZE = Czechia; DEU = Germany; DNK = Denmark; DOM = Dominican Republic; EGY = Egypt; ESP = Spain; EST = Estonia; FIN = Finland; FRA = France; FRO = Faroe Islands; GBR = United Kingdom; GHA = Ghana; GRC = Greece; HRV = Croatia; HUN = Hungary; IDN = Indonesia; IND = India; IRL = Ireland; IRN = Iran; ISR = Israel; ITA = Italy; JPN = Japan; KHM = Cambodia; KOR = South Korea; LBN = Lebanon; LTU = Lithuania; LUX = Luxembourg; LVA = Latvia; MAR = Morocco; MEX = Mexico; MLT = Malta; MNG = Mongolia; MYS = Malaysia; NLD = Netherlands; NOR = Norway; NZL = New Zealand; PER = Peru; POL = Poland; PRI = Puerto Rico; PRT = Portugal; ROU = Romania; RUS = Russia; SGP = Singapore; SVK = Slovakia; SVN = Slovenia; SWE = Sweden; THA = Thailand; TWN = Taiwan; UKR = Ukraine; USA = United States; VEN = Venezuela; VNM = Vietnam; ZAF = South Africa.  
Empty cells represent non-available information.
